# Immunoregulation of Glia after spinal cord injury: a bibliometric analysis

**DOI:** 10.3389/fimmu.2024.1402349

**Published:** 2024-06-13

**Authors:** Yi Huang, Rong Hu, Lei Wu, Kelin He, Ruijie Ma

**Affiliations:** ^1^ Key Laboratory of Acupuncture and Neurology of Zhejiang Province, The Third School of Clinical Medicine (School of Rehabilitation Medicine), Zhejiang Chinese Medical University, Hangzhou, China; ^2^ Department of Acupuncture, The Third Affiliated Hospital of Zhejiang Chinese Medical University, Hangzhou, China

**Keywords:** spinal cord injury, glial cells, immunoregulation, bibliometrics, global trends

## Abstract

**Objective:**

Immunoregulation is a complex and critical process in the pathological process of spinal cord injury (SCI), which is regulated by various factors and plays an important role in the functional repair of SCI. This study aimed to explore the research hotspots and trends of glial cell immunoregulation after SCI from a bibliometric perspective.

**Methods:**

Data on publications related to glial cell immunoregulation after SCI, published from 2004 to 2023, were obtained from the Web of Science Core Collection. Countries, institutions, authors, journals, and keywords in the topic were quantitatively analyzed using the R package “bibliometrix”, VOSviewer, Citespace, and the Bibliometrics Online Analysis Platform.

**Results:**

A total of 613 papers were included, with an average annual growth rate of 9.39%. The papers came from 36 countries, with the United States having the highest output, initiating collaborations with 27 countries. Nantong University was the most influential institution. We identified 3,177 authors, of whom Schwartz, m, of the Weizmann Institute of Science, was ranked first regarding both field-specific H-index (18) and average number of citations per document (151.44). Glia ranked first among journals with 2,574 total citations. The keywords “microglia,” “activation,” “macrophages,” “astrocytes,” and “neuroinflammation” represented recent hot topics and are expected to remain a focus of future research.

**Conclusion:**

These findings strongly suggest that the immunomodulatory effects of microglia, astrocytes, and glial cell interactions may be critical in promoting nerve regeneration and repair after SCI. Research on the immunoregulation of glial cells after SCI is emerging, and there should be greater cooperation and communication between countries and institutions to promote the development of this field and benefit more SCI patients.

## 1 Introduction

Spinal cord injury (SCI) is a devastating central nervous system (CNS) disease with high morbidity, disability, and cost, which is the leading cause of paralysis (1). The global crude incidence of SCI ranges from 12.1 to 57.8 per million (2). A cross-sectional survey showed that there are more than 750,000 patients with traumatic spinal cord injuries in China, with approximately 66,374 new cases occurring each year (3). The National Spinal Cord Injury Statistics Centre estimates that the first-year cost of a high tetraplegic patient exceeds $1.06 million (4). Spinal cord injuries not only lead to motor and sensory nerve dysfunction but are also associated with an increased risk of pulmonary infections, urinary tract infections, deep vein thrombosis, and a variety of diseases (5). Compared to non-SCI patients, SCI patients have lower immune cell function and higher rates of infection (6). SCI follows a series of pathological processes such as ischemia, edema, inflammation, apoptotic necrosis, axonal degeneration, and glial scarring (1). However, the pathological mechanisms remain unknown, and the role of different cell types in SCI remains controversial.

Immunoregulation after SCI is a complex and critical process involving multiple cellular and molecular interactions that are essential for the repair of SCI (7, 8). The immunomodulatory effects of SCI are mainly in the modulation of inflammatory responses, immune cell regulation, and neuroprotection (9). And glial cells play a pivotal role in the immunomodulation of the CNS (10, 11). Neuroglial cells consist of several subtypes, including three main types: microglia, astrocytes, and oligodendrocytes. They regulate the intensity, duration, and nature of immune responses by releasing immunomodulatory molecules and interacting with other immune cells, thereby maintaining the balance and health of the CNS, and affecting the prognosis of SCI (12–14). Although the correlation between glial cells and SCI has been reported, it is still important to explore and summarize the research related to the immunoregulation of glial cells after SCI to accurately grasp the direction of the research and find new therapeutic targets. Currently, limitations of the main treatment for SCI, like glucocorticoids (grade A prednisolone) and bionic material implantation, include high toxicity, limited efficacy, and high cost. Developing targeted immunomodulatory drugs can help improve patients’ quality of life, improve neurological regeneration and repair, and benefit more SCI patients.

Bibliometric analysis is an interdisciplinary study that uses mathematics and statistics to quantitatively analyze literature information to measure the impact, interrelationships, and trends of publications in a specific field (15). It is now widely used in various medical fields (16). Literature review refers to the summarization and synthesis of the content of previous literature within a predefined research framework. In contrast, bibliometrics provides a more intuitive and systematic analysis of publications in a quantifiable manner, which facilitates researchers in grasping the overall trends in the field. Several reviews explored the mechanisms involved in glial cell immunoregulation after SCI (17–19). However, summaries of recent studies are rare, and bibliometric analyses in this area are unavailable. To fill this gap, this study combined quantitative and qualitative analyses to explore future research directions and goals in glial cell immunoregulation after SCI.

## 2 Materials and methods

### 2.1 Data sources and search strategies

**Figure 1:**
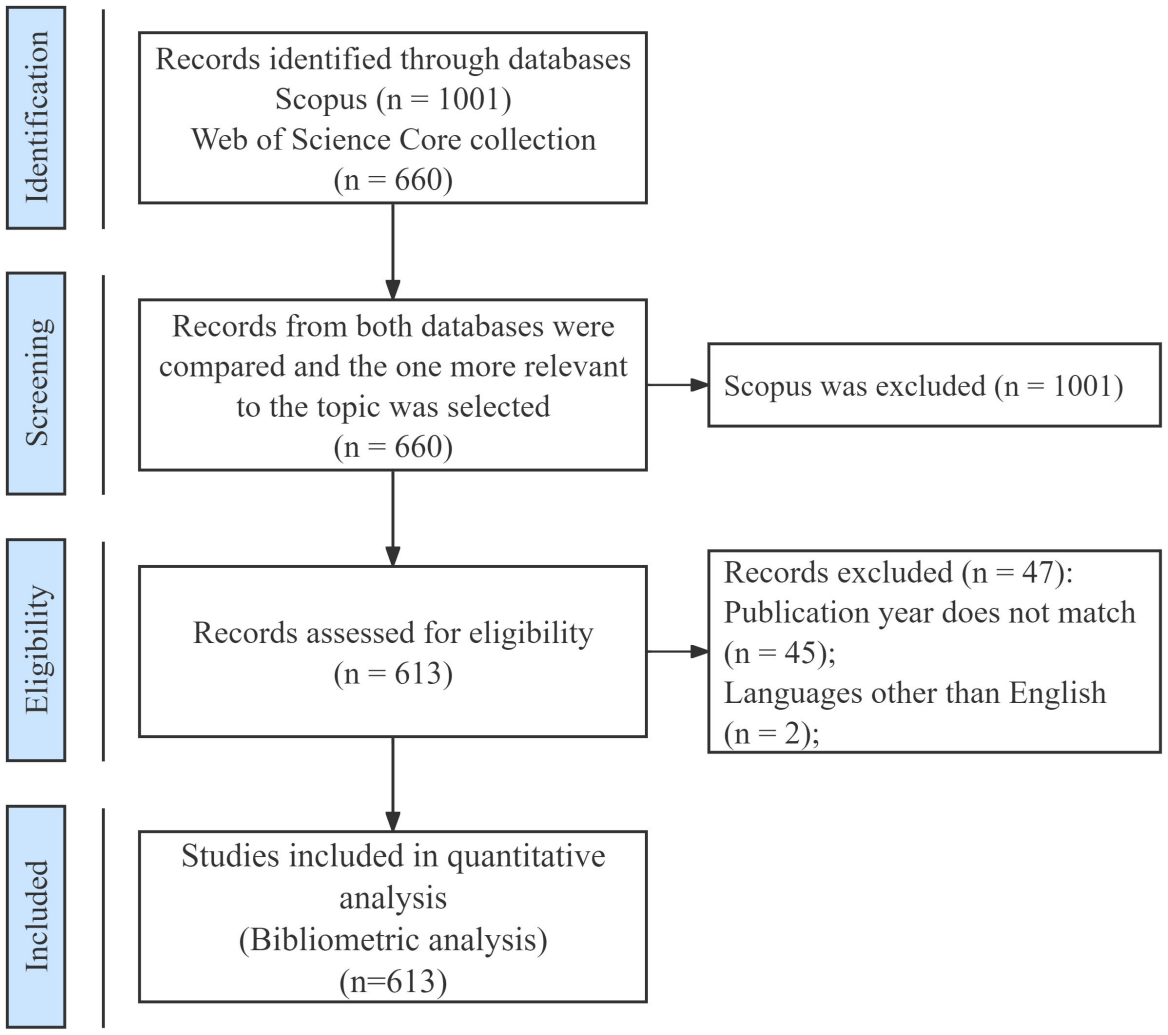
Flowchart of publication search and selection.

The Web of Science Core Collection (WoSCC) was searched for relevant English-language publications published from 2004 to 2023. The search strategy was as follows: (TS = (“spinal cord injury” OR “spinal cord injuries” OR “spinal cord trauma” OR “spinal cord contusion”) AND TS = (“immunity” OR “immunological” OR “immunology” OR “immune*”) AND TS = (“neuroglia” OR “glial cell” OR “astrocyte” OR “oligodendrocyte” OR “microglia” OR “ependymal cell”)). The screened and qualified 613 publications were downloaded as Full Record and Cited References and saved as “BibTex” and “Plain Text File” for further analysis. The specific process is shown in **Figure 1**.

### 2.2 Data analysis

The bibliometric analyses used four tools, namely R software (version 4.1.2), VOSviewer (version 1.6.19), Citespace (version 6.3.1), and the Bibliometric Online Analysis Platform (https://bibliometric.com/). Bibliometrix is a data visualization tool written in R for comprehensive bibliometric mapping analysis through the Biblioshiny application for aggregating publications and citations by country, institution, journal, and author (20). VOSviewer is a free Java-based software adapted to one-mode undirected network analysis for constructing national citation networks, author co-citation networks, and keyword co-occurrences (21, 22). Citespace is an information visualization software based on the theory of citation analysis, focusing on prospective data in the scientific literature (23). We used Citespace to detect keywords and references with high citation bursts. The Bibliometric Online Analysis Platform analyzes uploaded citations through a WEB-based service for analyzing international collaborations (24).

## 3 Results

### 3.1 Analysis of annual publications

**Figure 2:**
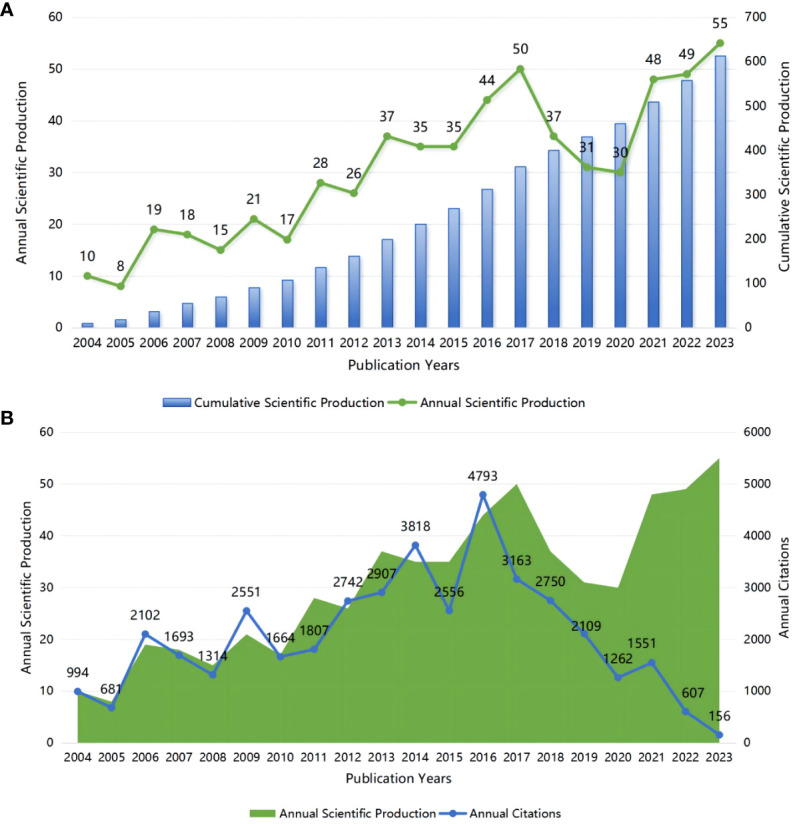
**(A)** Annual and cumulative scientific production; **(B)** Annual scientific production and annual citations.

A total of 613 papers related to glial cell immunoregulation after SCI were included, of which 425 were articles and 179 were reviews (**Figure 2A**). In the last 20 years, the number of relevant papers has increased steadily, with an average annual growth rate of 9.39%. Among them, the average annual number of publications from 2004 to 2013 was 19.9, showing a slow growth trend. Over the last decade, the number of papers has grown significantly, from 234 in 2014 to 613 in 2023, with an average of 40.4 per year. We further analyzed the annual citations. The average number of citations for publications was 67.24. The 21 relevant publications from 2009, with 2551 citations, indicate that the publications from that year received a great deal of attention from researchers (**Figure 2B**). So far in 2016, citations have generally trended in the opposite direction of publications.

### 3.2 Analysis of countries

**Figure 3:**
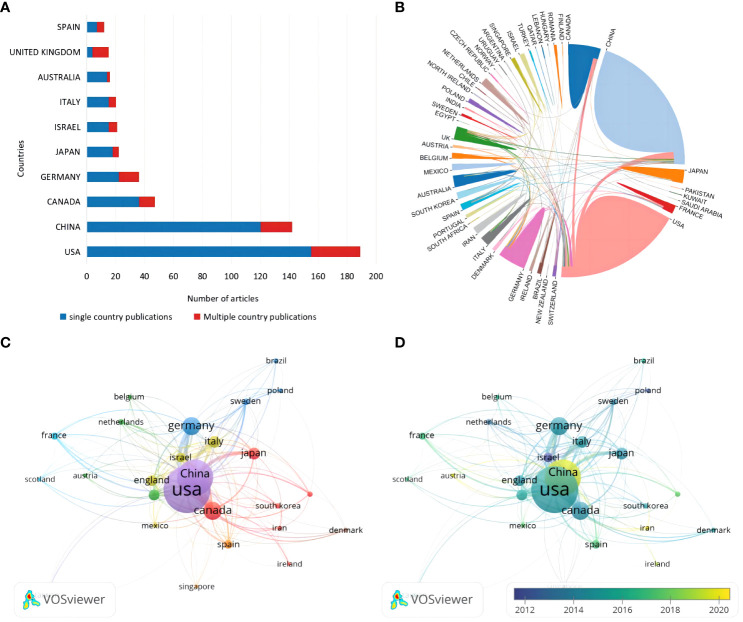
**(A)** Corresponding authors’ country distribution. **(B)** International cooperation analysis. The area occupied by countries in a circle is directly proportional to related publications, and the thickness of the connecting lines between countries reflects the closeness of cooperation. **(C)** Citation network visualization of countries. Each country is represented as a node, and the node size is proportional to the number of references. **(D)** Citation overlay visualization for countries. Each country is represented as a node, the node size is proportional to the number of citations, and the lines between the nodes are given different colors according to the color gradient of each year in the legend.

A total of 36 countries, according to the nationality of the corresponding author, are involved in the field of glial cell immunoregulation after SCI. The United States (U.S.) had 189 publications (30.83%), followed by China (23.16%) and Canada (7.67%), while the remaining countries had less than 40 relevant ones (**Figure 3A**). Single country publications (SCP) denote publications co-authored by authors of the same nationality while multiple country publications (MCP) denote publications co-authored by authors of several nationalities. Although the U.S. accounted for only 23.45% of the total MCP, it was still the most active country in international cooperation, working with 27 countries (**Figure 3B**). In particular, China was the most closely linked, with 29 times of cooperation. The United Kingdom (UK) ranked ninth in publications, with MCP rates of over 70%, suggesting a high level of international influence.

A total of 26 countries were included in the analysis when using Vosviewer for citation analysis by limiting the minimum number of citations to five (**Figure 3C**). Each node represents one country, and the thickness of the connecting lines reflects the total link strength (TLS). Not surprisingly, the country’s TLS ranking was directly proportional to the number of publications. The U.S. ranked first with a TLS of 1,047 and 20,044 total citations. China, Australia, and Iran emerged as promising countries in this field in the last five years (**Figure 3D**). Among them, China has the highest emerging research momentum.

### 3.3 Analysis of institutions

**Figure 4:**
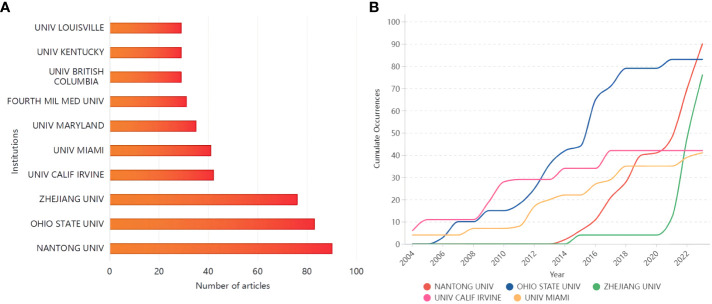
**(A)** The top ten institutions in terms of the number of publications; **(B)** Top five institutions’ production over time.

The U.S. accounted for six of the top ten most relevant institutions (**Figure 4A**). Nantong University started late in the field of glial cell immunomodulation after SCI, but has risen rapidly in the past three years, publishing as many as 90 relevant papers (**Figure 4B**). In contrast, Ohio State University started earlier and has seen a steady increase in publications, ranking second in the number of articles issued (13.53%).

### 3.4 Analysis of authors

**Figure 5:**
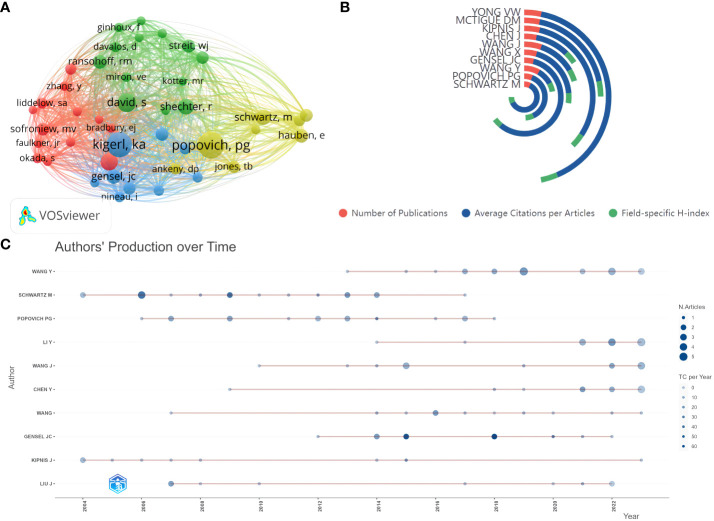
**(A)** Author co-citation analysis network visualization. Each node represents an author, the node’s size is proportional to the number of citations, and the node’s color represents different research topics. A link between two nodes indicates a co-reference relationship. **(B)** Top five authors’ publications, average number of citations per article, and field-specific H-index. **(C)** Authors’ production over time. The red line between the circles is the timeline of relevant publications by that author, the circle diameter is proportional to the number of publications, and the saturation of the circle color is proportional to the total citations per year.

Based on the author lists of relevant publications, excluding duplicates, 3177 authors were involved in writing articles related to glial cell immunoregulation after SCI. With a threshold of no less than 50 citations, 43 authors were included in the co-citation analysis (**Figure 5A**). Those with small node spacing have similar themes and are assigned to the same cluster (25). The author with the highest TLS is Popovich, PG of The Ohio State University, followed by Kigerl, ka of The Ohio State University, and Schwartz, m of the Weizmann Institute of Science, which side-steps author influence. Based on this dataset calculation, Schwartz, m was the author with the highest field-specific H-index (18) and the highest average number of citations per document (151.44) (**Figure 5B**). In the last 20 years, he has published 18 related papers with 2,726 total citations. John C Gensel of the University of Kentucky published highly cited papers in 2015 and 2018, with over 50 total citations per year, making him highly influential in the field (**Figure 5C**). As can be seen, research on glial cell immunoregulation after SCI has been on the rise in the last decade, and more and more researchers have begun to focus on this topic.

### 3.5 Analysis of journals

The Journal Citation Report (JCR) is a journal analysis tool provided by Corevision that facilitates researchers to quickly learn about authoritative journals in their discipline (26). A total of 227 academic journals published articles on glial cell immunomodulation after SCI, of which 46.12% were in the first and 35.44% in the second quartile. The Journal of Neuroinflammation, Experimental Neurology, Glia, Journal of Neurotrauma, and Brain Behavior and Immunity were the top five in paper production, with 21% of the total number of publications, and widely recognized in the field (**Table 1**). Among them, Glia published 27 articles and ranked first in total citations with 2,574, which is highly influential.

**Table 1:** Top 10 most relevant journals.

Journals	Articles	Total Citations	JCR (2022)	IF (2022)	Fields
Journal of Neuroinflammation	35	2000	Q1	9.3	Immunology; Neurosciences
Experimental Neurology	31	1987	Q2	5.3	Neurosciences
Glia	27	2574	Q1	6.2	Neurosciences
Brain Behavior and Immunity	20	1500	Q1	15.1	Immunology; Neurosciences; Psychiatry
Journal of Neurotrauma	19	772	Q2	4.2	Clinical Neurology; Critical Care Medicine; Neurosciences
Journal of Neuroscience	18	2270	Q1	5.3	Neurosciences
Neural Regeneration Research	12	287	Q1	6.1	Cell Biology; Neurosciences
PLoS One	12	620	Q2	3.7	Multidisciplinary Science
Frontiers in Cellular Neuroscience	11	523	Q1	5.3	Neurosciences
Journal of Neuroimmunology	11	641	Q3	3.3	Immunology; Neurosciences

JCR, Journal Citation Report; IF, impact factor; Q, Quartile in Category.

### 3.6 Analysis of research hotspots

#### 3.6.1 Analysis of total citation frequency

Total citation frequency analysis is a widely used tool for citation analysis, which can intuitively reflect the academic influence of an article in a specific field (27). The top 10 articles all had more than 500 citations, and 50% of these studies focused on inflammatory responses (**Table 2**). The article “Neuroinflammation: The Devil is in the Details,” published in 2016, had the most citations with 789 (28). The total citations often correlate with the year of publication, and total citations per year are analyzed to help circumvent this problem. A review published in 2019 titled “Traumatic Spinal Cord Injury: An Overview of Pathophysiology, Models and Acute Injury Mechanisms” ranked first with an annual average of 101.83 citations, providing a comprehensive overview of the pathophysiology of SCI (19). In addition, the article elaborated on the role of astrocytes, microglia, and others in the innate immune response to SCI.

**Table 2:** Top 10 articles in total citations.

Rank	Title	Journal	Year	Total citations	Total citations per year
1	Neuroinflammation: the devil is in the details	J Neurochem	2016	789	87.67
2	Innate immunity in the central nervous system	J Clin Invest	2012	718	55.23
3	Neuroinflammation and Central Sensitization in Chronic and Widespread Pain	Anesthesiology	2018	670	95.71
4	Traumatic Spinal Cord Injury: An Overview of Pathophysiology, Models and Acute Injury Mechanisms	Front Neurol	2019	611	101.83
5	Infiltrating blood-derived macrophages are vital cells playing an anti-inflammatory role in recovery from spinal cord injury in mice	PLoS Med	2009	594	37.13
6	Cell transplantation therapy for spinal cord injury	Nat Neurosci	2017	569	71.13
7	Astrocytes: Key Regulators of Neuroinflammation	Trends Immunol	2016	568	63.11
8	Activated microglia contribute to the maintenance of chronic pain after spinal cord injury	J Neurosci	2006	517	27.21
9	The role of markers of inflammation in traumatic brain injury	Frontiers in Neurology	2013	509	42.42
10	Macrophage activation and its role in repair and pathology after spinal cord injury	Brain Res	2014	501	50.10

#### 3.6.2 Analysis of reference citation bursts

**Figure 6:**
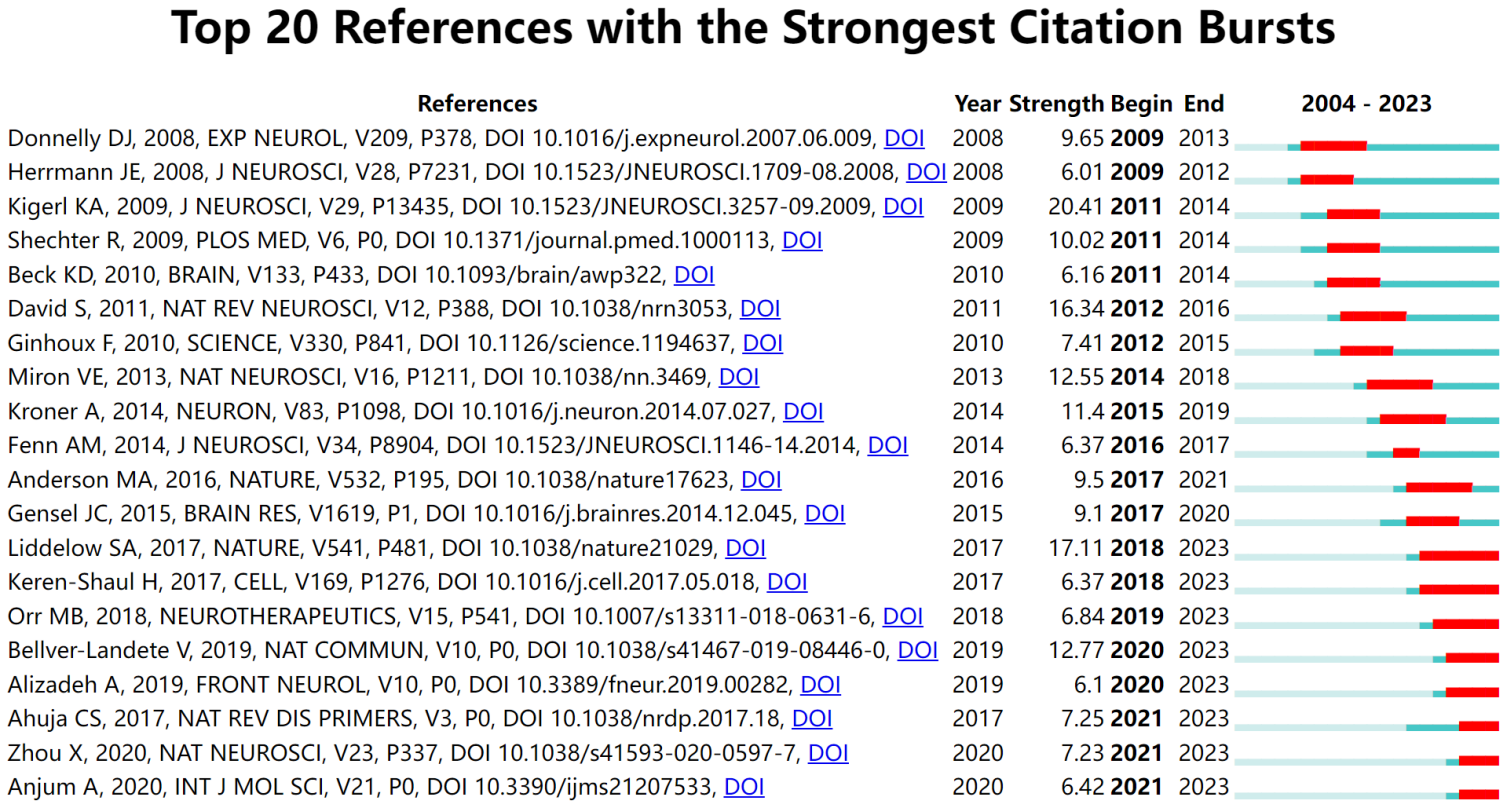
Top 20 references with the strongest citation bursts. The light green line segment indicates the pre-publication period of the article, the dark green line segment indicates the post-publication period of the article, and the red line segment is the duration of the article’s burst.

Citation burst analysis for detecting references with citation spikes is essential for tracking and capturing hotspots (29). The article “Identification of two distinct macrophage subsets with divergent effects causing either neurotoxicity or regeneration in the injured mouse spinal cord” published in the Journal of Neuroscience in 2009, has the highest burst value, at 20.41 (**Figure 6**). This study demonstrated that polarized differentiation of microglia and infiltrating blood monocytes towards M2 or “alternatively” activated macrophage phenotypes could promote CNS repair while limiting secondary damage (30). In addition, the citation burst of five publications focusing primarily on microglia, including “Microglia and macrophages promote corralling, wound compaction and recovery after spinal cord injury via Plexin-B2,” continues, suggesting that such studies are potentially at the forefront of research on glial cell immunoregulation after SCI.

#### 3.6.3 Analysis of keyword co-occurrence

**Figure 7:**
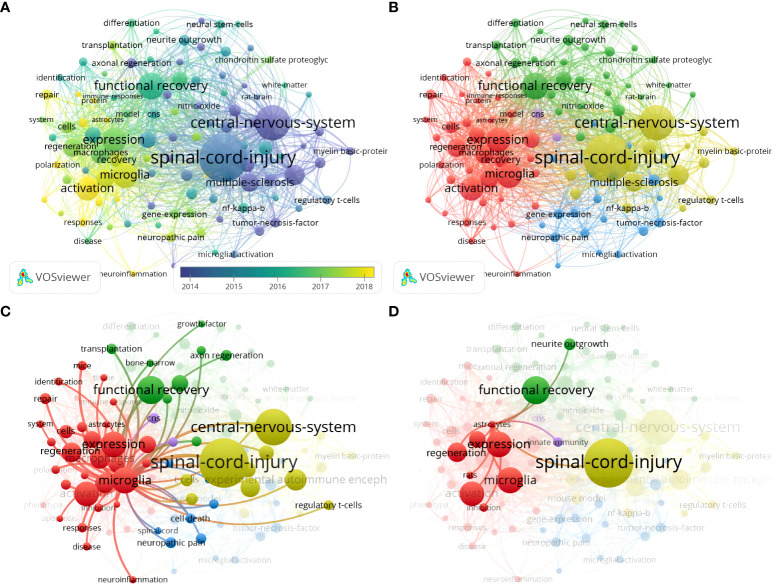
**(A)** Keyword co-occurrence analysis overlay visualization; **(B)** Keyword co-occurrence analysis network visualization; **(C)** Keyword co-occurrence analysis network visualization centered on microglia; **(D)** Visualization of a keyword co-occurrence analysis network centered on astrocytes.

Keyword co-occurrence analysis identifies emerging research themes and their relevance by counting the frequency and co-occurrence of keywords in the literature (31). With a threshold of not less than ten occurrences, 104 keywords out of 1756 met the criteria. The keywords “central nervous system,” “spinal cord injury,” “tumor necrosis factor,” and “regulatory t cells” are bluish, which represents early research themes (**Figure 7A**). In contrast, “microglia,” “activation,” “macrophages,” “astrocytes, “ and “neuroinflammation” are yellowish, which represents recent hot topics and may still be future research frontiers. Based on the TLS and direction, the keywords clustered into four different colored clusters, and the representative keywords were “microglia,” “spinal cord injury,” “functional recovery,” and “central nervous system,” which were consistent with the research field (**Figure 7B**). In addition, “microglia” was also widely associated with keywords of other clusters like “immune response,” “axon regeneration,” and “cell death,” which received high attention (**Figure 7C**). In addition to being associated with “microglia”, “astrocytes” were strongly associated mainly with “neurite outgrowth” and “regeneration” (**Figure 7D**). “Oligodendrocyte” did not appear in the keyword co-occurrence.

#### 3.6.4 Analysis of keyword citation bursts

**Figure 8:**
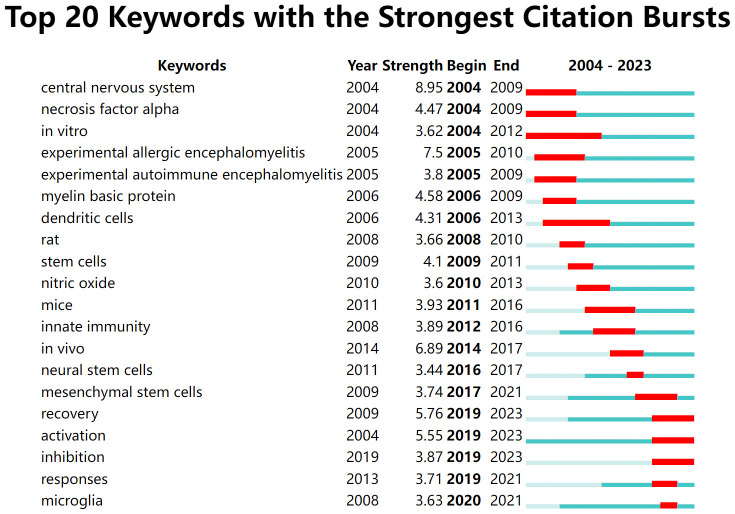
Top 20 keywords with the strongest citation bursts. The light green line segment indicates before the keyword appears, the dark green line segment indicates after the keyword appears, and the red line segment is the duration of the keyword citation burst.

Keyword citation bursts can clearly show the research hotspots and their changing trends to provide direction for subsequent research in the field (32). “Central nervous system” (strength: 8.95), “necrosis factor alpha” (strength: 4.47), and “*in vitro*” (strength: 3.62) were the earliest highly cited burst keywords in the last 20 years (**Figure 8**). The citation burst for “dendritic cells” (strength: 4.31) lasted from 2006 to 2013. The keywords that have been bursting in the last five years were “recovery” (strength: 5.76), “activation” (strength: 5.55), “inhibition” (strength: 3.87), “responses” (strength: 3.71), and “microglia” (strength: 3.63), which are consistent with the keyword co-occurrence results.

## 4 Discussion

In this study, we conducted a bibliometric analysis of the immunoregulation of glial cells after SCI using WoSCC to gain a comprehensive understanding of global research trends and hotspots for researchers in this field.

### 4.1 Global trends in glial cell immunoregulation after spinal cord injury

The number and citation of annual research outputs are valuable indicators of the development of an academic field. Over the past two decades, there has been a steady increase in articles on glial cell immunoregulation after SCI. A previous bibliometric study focusing only on SCI found a similar trend (33). A potential reason for the expansion of research may be the growing recognition of the role of glial cell immunoregulation after SCI and, consequently, increased research funding in this field (17). In the last ten years, there was a surge of papers in this field compared to the previous period, while the annual citation frequency showed an opposite trend, suggesting that research on glial cell immunoregulation after SCI is emerging, but the overall level of publications still needs to be improved.

Assessing the closeness of cooperation between countries can help guide scientific research activities and promote potential cooperation opportunities for other groups. In this bibliometric analysis, most of the relevant articles were co-published by corresponding authors from multiple countries, such as the U.S., China, Canada, and Germany. Similar writing patterns have been found in bibliometric studies in medical fields such as stroke, lymphoma, and Parkinson’s disease (34–36). A country’s medical research output depends heavily on its economy and expenditure on health care. According to data released by the Statista Research Department in 2024, the U.S. has more than $4.4 trillion in annual health expenditures, more than all other countries (37). That may explain the top ranking of the U.S. in terms of number of publications, total citations, and international collaborations.

The number of articles published by an institution can reflect its activities and contributions in the subject area. Nantong University in China, a rising star in the field, has published nearly 50 relevant articles in the last three years, which were closely related to the development of its affiliated Key Laboratory of Neural Regeneration. Although Canada ranked third in publications, only one research institution was in the top ten, suggesting an absence of specialized research institutions. High-level organizations and groups lead the way in research into glial cell immunoregulation after SCI, and further research at these institutions will ensure the continued development of the field in the future.

Unlike the conventional H-index, the field-specific H-index is calculated based on a dataset filtered by research topics, which is more conducive to the reflection of the scientific productivity and reputation of researchers in specific research directions. According to the authors’ analysis, the field-specific H-index of Schwartz, m, a neuroimmunologist, was significantly lower (18) than the H-index on the Web of Science display (38). It does not mean that this author’s influence has diminished, but because her research is broad, focusing more on studying the relationship between the immune system and the brain, in addition to glial cell immunoregulation after SCI. John C Gensel of the University of Kentucky’s highly cited papers in 2015 and 2018 were related to their research types, which were reviews focusing on the roles of neuroglia and inflammatory cells in the repair and pathology after SCI and the corresponding therapeutic strategies, respectively, and significantly contributing to the exchange of scientific research (39, 40).

Keeping an eye on journal publication trends and research areas helps researchers better track research developments and select target journals. More than 80% of the 227 academic journals were in the first or second quartile, indicating that the relevant published content was well worth reading. Most of the top ten journals were in the neuroscience track, with journals in psychiatry, critical care medicine, and cell biology also involved. Among them, Glia ranked first with 2,574 total citations, publishing research on the mechanisms of neuroglia’s role in myelin regeneration, neuroprotection, and inflammatory response in SCI (41–43).

### 4.2 Hot spots and research development forecasts

**Figure 9:**
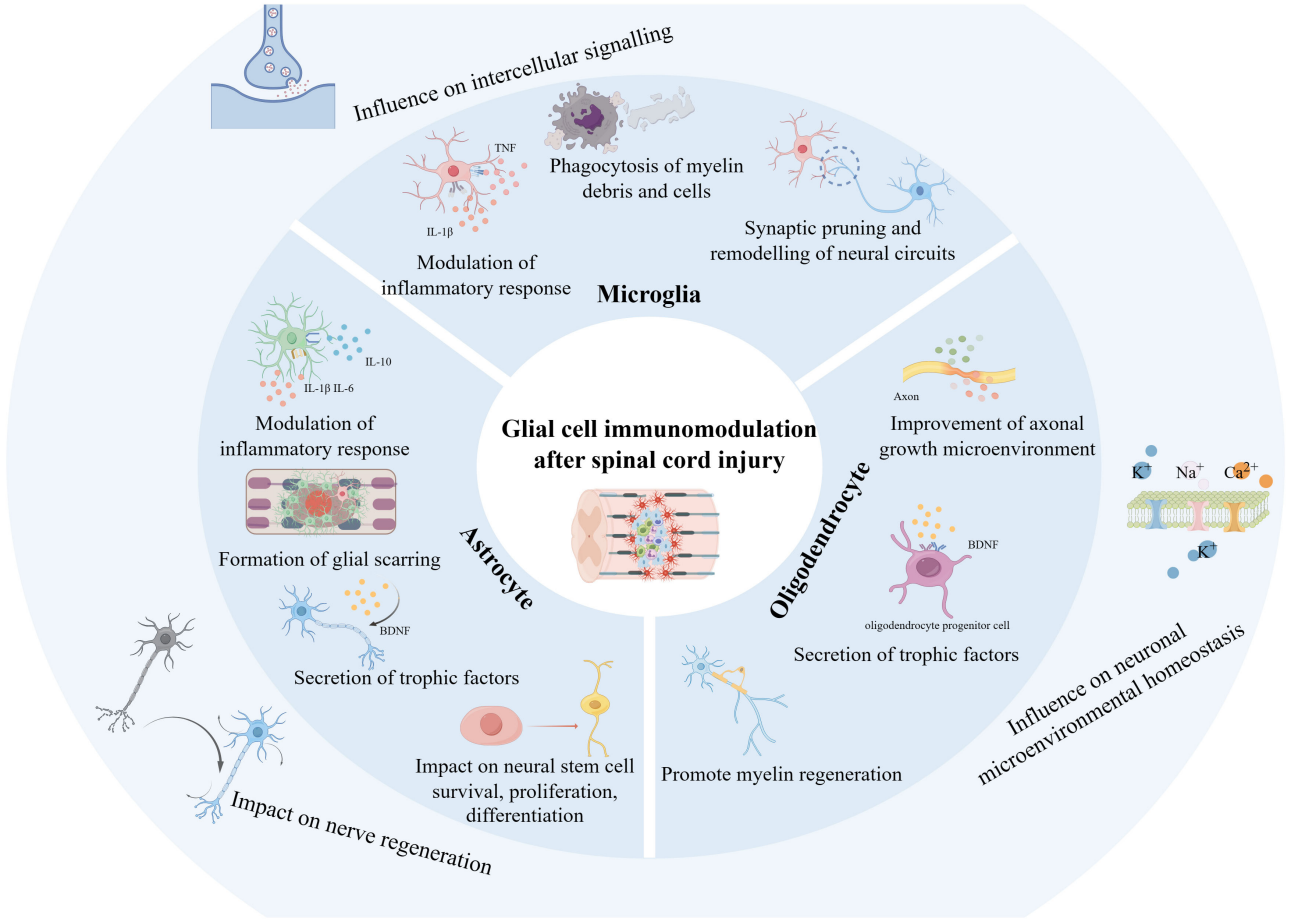
Schematic representation of glial cell immunoregulation after SCI based on the study. The diagram was generated by BioRender.com.

High-frequency keywords are commonly used to accurately reveal the main research directions and hotspots in specific fields within a certain period. Combined with burst detection, they can help researchers rapidly capture research trends from numerous studies. We found from the keyword co-occurrence in the field of glial cell immunoregulation after SCI that “microglia,” “activation,” “macrophages,” “astrocytes,” and “neuroinflammation” represented recent hot topics and are likely to remain at the forefront of research shortly. The keyword co-occurrence network visualization clustered the keywords into four different clusters based on TLS and direction, “microglia,” “spinal cord injury,” “functional recovery,” and “central nervous system.” Among them, “microglia” was widely associated with keywords of other clusters, such as “immune responses,” “axonal regeneration,” “cell death,” etc., and enjoyed great popularity. In addition to being strongly associated with “microglia,” “astrocytes” were mainly related to “neurite outgrowth,” and “regeneration” primarily. Combined with keywords and references that continue to show high bursts, we analyzed current research hotspots and predicted future research trends (**Figure 9**).

#### 4.2.1 Microglia immunoregulation may be critical for axonal regeneration after spinal cord injury

Microglia play an influential role in SCI throughout the pathologic process and nerve repair. The importance of microglia immunoregulation for axon regeneration after SCI has also received increasing attention (44, 45). Microglia are intrinsic immune cells of the CNS, responsible for monitoring and clearing pathogens, damaged cells, and cellular debris. They can induce immune responses by recognizing and activating relevant pathways such as P38/MAPK, NF-KB, and PI3K/Akt/mTOR through Toll-like receptors and others. In addition, it can recruit inflammatory cells by secreting cytokines such as TNF-a, IL-1B, IL-6, and IL-12, causing a cascade response of inflammation (46). After SCI, microglia respond rapidly by secreting and releasing inflammatory mediators and cytokines, such as ROS, TNF-a, and CCL2, which exacerbate the inflammatory response and lead to more immune cells infiltrating the injury site, further amplifying the immune response (47). Critically, this array of immunomodulatory molecules plays a crucial role in the control of secondary damage and neural regenerative repair after SCI by regulating the active migration and proliferation of T-cells, B-cells, and other immunocytes, and controlling the intensity and duration of the inflammatory response (18).

What’s more, during SCI, on the one hand, hyperactivation of microglia exacerbates inflammatory response and neurotoxicity, while on the other hand, microglia are significant in axonal regeneration after injury through phagocytosis to remove myelin debris and cellular debris, as well as by interacting with other immune cells and neurons, which collectively regulate the repair process after injury (45, 48–50). Experimental studies have shown that after SCI, microglia promote oligodendrocyte generation and axonal regeneration through MyD88, phagocytosis of debris degrading myelin sheaths, and secretion of a variety of growth factors and neurotrophic factors (e.g., nerve growth factor and brain-derived neurotrophic factor) (51–53). Microglia activated after SCI also release exosomes. These microglia-derived exosomes are enriched in miR-151-3p, a molecule that can deliver to neurons and target-bound to Trp53, which further inhibits the p53/p21/CDK1 signaling pathway, reduces neuronal apoptosis, and promotes axon regeneration and repair of neurological function after injury (54). Therefore, regulating the activity of microglia and keeping them in an appropriately activated state is essential for promoting axonal regeneration.

In conclusion, microglia are involved in axonal regeneration during SCI by regulating the immune response, promoting neurite growth and synapse formation, and removing inhibitory molecules, which provide essential support for spinal cord repair and regeneration, and together, these mechanisms constitute a complex immunoregulatory network of microglia after SCI (55). Future studies will further reveal the specific mechanism of microglia’s role in SCI repair and provide ideas for developing new therapeutic strategies.

#### 4.2.2 Astrocyte immunomodulation may be essential for neuronal regeneration after spinal cord injury

Astrocytes are one of the most abundant glial cell types in the CNS, which provide support and protection to neurons under normal physiological conditions and play an influential role in pathological states such as SCI (56–59). Following injury, astrocytes respond rapidly and participate in immunomodulatory processes as the primary source of chemokines engaged in inflammatory cell recruitment (60–62). Astrocytes interact with macrophage migration inhibitory factors by expressing a range of receptors involved in innate immunity, such as Toll-like receptors, nucleotide-binding oligomerization domains, double-stranded RNA-dependent protein kinases, scavenger receptors, and components of the complement system (63), which inevitably exacerbate the neuropathological changes that occur after CNS injury (64). In addition, astrocytes recruit inflammatory cells to the injured site by interacting with CD74 receptors, activating JNK to release the chemokine CCL5, which further promotes the migration of macrophages toward the lesion site, thereby affecting the repair of the injured spinal cord (65).

In addition, astrocytes create a favorable neuronal microenvironment for tissue repair and neuronal synapse regeneration in injured tissues through interactions with other immune cells (66). Neuronal regeneration is the regrowth and reattachment of axons or dendrites of damaged neurons and is essential for restoring nerve function after SCI. Astrocytes can directly promote the growth and regeneration of neural synapses by releasing substances such as growth factors, neurotrophic factors, and extracellular matrix molecules (67–69). In addition to providing the necessary nutritional support, these molecules can guide the growth of neural synapses in the right direction, thus re-establishing neural connections (70). Astrocytes also promote neuronal regeneration indirectly by modulating synaptic transmission and plasticity (71). They affect signal transmission and connection strength between neurons by releasing neurotransmitters and modulating the expression of postsynaptic receptors (72). This modulatory effect helps to enhance the plasticity and adaptability of damaged neurons, thereby promoting the regeneration of neuronal synapses and functional recovery.

#### 4.2.3 Glial cell interactions may be vital in promoting neurological repair after spinal cord injury

Interactions between glial cells play a significant role in the repair of neurological function after SCI (73, 74): on the one hand, microglia can regulate the activity and function of astrocytes, affecting their ability to release growth factors and neurotrophic factors and indirectly influencing the survival of oligodendrocytes; on the other hand, astrocytes can also affect microglia activity and phagocytosis through the release of immunomodulatory molecules (38). This interaction helps to create a microenvironment that facilitates nerve regeneration and repair. They may act synergistically to modulate the inflammatory response and attenuate tissue damage and neuronal death (75). In addition, they may also interact with each other through the secretion of growth factor and neurotrophic factor, etc., which collectively promote neuronal survival, axonal regeneration, and synaptic plasticity, thus contributing to the restoration of neurological function (76, 77).

Activated M1 microglia after SCI is one of the essential influences on oligodendrocyte necrotic apoptosis (78), and reduction of pro-inflammatory microglia expression significantly reduces oligodendrocyte death after SCI, decreases neuronal loss and demyelination, and improves functional recovery after SCI (79). Activated microglia can also promote a reactive astrocyte-mediated tissue necrosis response through the TLR/MyD88 signaling pathway, inducing cavity formation and exacerbating secondary injury in SCI (80). Furthermore, after SCI, interferon-induced transmembrane protein 1 is rapidly increased in infiltrating leukocytes, activated microglia, and astrocytes, impeding the recovery of neurological function after injury (81). Microglia can also positively affect astrocytes and oligodendrocytes. After SCI, microglia are activated and produce type I interferon, which binds to the heterodimeric receptor IFNAR and promotes the transcriptional activation of heterodimeric STAT1/2 nuclear translocations and ISGs, which constitutes a complete type I interferon signal, initiating a signaling cascade response that further enhances neuronal and astrocytic activity (82). In addition, microglia secrete TGFB and IGF after SCI, reversing type A1 astrocyte polarization, which attenuates immune infiltration, promotes neuronal and oligodendrocyte survival, and enhances recovery of neurological function after injury (83). Microglia can also inhibit the PY1 receptor of astrocytes through purinergic signaling, attenuate the effects of inflammatory factors such as IL-6, IL-1B, and TNFα on astrocytes, promote the formation of glial scars in the injured area, reduce leukocyte infiltration, and promote the survival of neurons as well as oligodendrocytes to a certain extent, thus contributing to the repair of neurological function after injury (84). Some studies have also shown that astrocytes activated after SCI release the inflammatory mediator chemokine CCL2, which promotes microglia activation and recruitment when combined with CCR2, prompting the release of IL-1β from microglia, which further exacerbates neuronal apoptosis and is detrimental to post-injury nerve repair and regeneration (85).

Nerve regeneration is critical for functional repair after SCI (86, 87). The glial cells around the injury are activated immediately after SCI and play an important role in nerve regeneration and repair by releasing inflammatory mediators, chemokines, etc., which act on immune cells. What other complex pathologic responses are involved in this process are worthy of in-depth study, e.g., What mechanisms drive the release of these cytokines from glial cells? Is the disruption of the blood-spinal cord barrier related to this process? Thus, further revealing how different glial cells regulate neuroimmune mechanisms after SCI and how different glial cells interact with each other is necessary to provide more possibilities for potential therapeutic targets and rehabilitation of SCI.

### 4.3 Strengths and limitations

This bibliometric study provided a systematic analysis of the basic situation, global trends, and research hotspots of glial cell immunoregulation after SCI from a visual perspective. The research findings were objective and accurate and can provide a comprehensive guide for scholars already working or wishing to work in the field. Still, several limitations exist in this study. Firstly, only publications from the WoSCC were included, excluding non-English language databases, although the database is reliable and contains a broadly representative collection. Secondly, there might be inconsistencies in the names of some institutions or journals at different times. Third, this analysis did not include publications outside of 2004-2023 because the database remains open.

## 5 Conclusion

This study investigated the immunoregulation of glial cells after SCI by bibliometric analysis, covering 2004 to 2023. Compared to other review articles, the contribution of this study is evident in the visualization that reveals the countries, institutions, authors, journals, popular keywords, and references that have had a significant impact on the field. Our study highlights the potential role of glial cell immunoregulation in SCI repair and identifies glial cells as a potential therapeutic target for SCI. There is no specific treatment for SCI, and our study suggests that immunoregulation of the interactions between microglia, astrocytes, and glial cells may be the key to promoting neurologic repair after SCI. Research on the immunomodulation of glial cells during SCI is emerging, and there should be greater cooperation and communication between countries and institutions to promote this field.

## Data availability statement

The original contributions presented in the study are included in the article/supplementary material. Further inquiries can be directed to the corresponding author.

## Author contributions

YH: Writing – review & editing, Writing – original draft, Methodology. RH: Writing – original draft, Data curation. LW: Writing – original draft, Project administration. KH: Writing – original draft, Software, Supervision, Resources. RM: Writing – review & editing, Resources, Project administration, Methodology, Funding acquisition.
